# Mirabegron as a Medical Expulsive Therapy for 5-10 mm Distal Ureteral Stones: A Prospective, Randomized, Comparative Study

**DOI:** 10.5152/tud.2022.22014

**Published:** 2022-05-01

**Authors:** Mohamed Abdel-Basir Sayed, Ahmed Mohamed Moeen, Hesham Saada, Anmar Nassir, Abdulmalik Tayib, Rabea Ahmed Gadelkareem

**Affiliations:** 1 Endourology Unit, Assiut Urology Hospital, Assiut University, Assiut, Egypt; 2 Department of Surgery, King Abdullah Medical City, Makkah, Saudi Arabia; 3 Department of Surgery, Umm Al Qura University, Makkah, Saudi Arabia; 4 King Abdulaziz University Hospital and International Medical Center, Jeddah, Saudi Arabia

**Keywords:** Distal ureteral stones, medical expulsive therapy, mirabegron

## Abstract

**Objective:**

The aim of this study was to assess the efficacy and safety of mirabegron as a medical expulsive therapy in patients with distal ureteral stones of 5-10 mm size.

**Material and methods:**

A prospective, comparative study included 96 patients with radiopaque distal ureteral stones of 5-10 mm who were randomly allocated and treated by medical expulsive therapy in 2 groups from January 2019 to December 2020. Patients in group A received only ketorolac 30 mg/day for 5 days, then on demand. Patients in group B received mirabegron 50 mg/day for 4 weeks plus ketorolac 30 mg/day like in group A. The stone expulsion rate was the primary outcome.

**Results:**

There were no significant differences regarding age, gender, body mass index, laterality, degree of hydronephrosis, and stone size. After 4 weeks, stone expulsion rate was 52.1% for group A versus 89.6% for group B (*P* < .001). The median (range) of time to stone expulsion was 14 (13-23) and 7 (3-16) days for groups A and B, respectively (*P* = .004). The medians (range; interquartile range) of episodes of renal pain (1 (0-2; 1) vs. (0-2; 2); *P* < .001) and extra analgesic ampoules (1 (0-7; 4) vs. 0 (0-2; 0) vials; *P* < .001) were significantly higher in group A than those in group B, respectively. In multivariate analysis, only medical expulsive therapy (*P* < .001) and stone size (*P* < .001) were independent predictors of stone expulsion rate.

**Conclusion:**

Mirabegron is an effective and safe medical expulsive therapy agent in patients with 5-10 mm distal ureteral stones.

**Main Points**

- Mirabigron plus ketorolac is superior to ketorolac alone as a medical expulsive therapy (MET) for distal ureteral stones (DUSs) of 5-10 mm.
- It provides a high stone expulsion rate (SER) between the second and fourth weeks up to 90%.
- It reduces the time to stone expulsion, episodes of renal colic, and the need for analgesia.
- Mirabegron may be associated with a low complication rate as a MET for DUSs.
- The modality of MET and stone size are independent predictors of the SER.

## Introduction

Ureteral stones represent about 20% of all urinary stones and 70% of them are located at the distal ureters at the time of diagnosis.^1^ Spontaneous passage of ureteral stones is relative to the stone size and is most possible for those less than 5 mm.^2^ Larger sizes are mostly symptomatic and indicate prescription of medical expulsive therapy (MET) including analgesics and ureteral dilating agents.^3,4^ Despite the evolving controversy about the presence of a strong evidence for the benefit of MET, the latter is still seen more effective and commonly used for distal ureteral stones (DUSs).^1,3^ Spontaneous stone expulsion depends on some factors such as stone size, configuration and location, spasm of ureteric smooth muscles, edema in the ureter, and anatomic structures.^5^ To accelerate the expulsion and reduce the complications of ureteral stones, many agents have been tried as MET including alpha-adrenergic blockers,^6^ calcium channel blockers,^7^ prostaglandin synthesis inhibitors,^8^ phosphodiesterase type-5 inhibitors,^9^ and steroids.^10^ Nowadays, alpha-adrenergic blockers are the most frequently used, efficient, and preferable MET agents in clinical practice. In contrast, many other agents have shown limited effects on the ureteral smooth muscles.^3,6^ The adverse effects of MET agents and the need for surgical intervention are unfavorable outcomes in variable proportions of patients. So, seeking the introduction of novel and more effective agents with lower complication profiles is still warranted.^3^

It has been shown that beta-3 adrenoreceptors (β3AR) are detected in the ureteral smooth muscles, mediating the adrenergic stimulation for ureteral relaxation.^11^ Moreover, urothelium and the interstitial cells themselves express β3AR more than the ureteral smooth muscle. This indicates that β3AR takes part in the dynamics of the ureter.^3,11^ Mirabegron is a selective β3AR agonist which has recently been introduced as a new MET agent. Supposedly, it may serve as an effective and safe alternative for the previously known agents of MET, which have different pathways of actions.^12^ In the current study, we examined the efficacy and safety of mirabegron as MET agent in patients with DUSs.

## Material and Methods

### Study Design

This is a prospective, randomized studying of adult patients with 5-10 mm radiopaque DUSs who were treated in our hospital, from January 2019 to December 2020. Radiologically, a DUS was defined as a stone located in the lower third of the ureter, distal to the level of the lower border of the sacroiliac joint down to the ureteral orifice level. Patients were assigned to have 1 of the 2 potential modalities of conservative management (groups A and B) using a computer-generated randomization method (JMP, version 12.0.1; SAS Institute, Cary, NC, USA). Two authors (MAS and RAG) were responsible for the patient’s assignment and revealing to the managing urologist to prescribe therapy.

### Population

Considering the rate of DUSs and previous similar studies,^12,13^ a sample size of 92 patients was calculated to provide a study power of 80% (type II error 0.2), a confidence level of 95% (type I error 0.05), and a threshold of significance of 0.05 using Epi Info™, version 3.5 software. A percentage of 5% extra patients was planned to compensate for the potential lost-to-follow-up patients.

Considering a possibility of lost follow-up patients, 96 patients with DUSs of 5-10 mm were included in the current study as 48 patients in each group. We excluded patients with urinary tract infection (UTI), multiple or bilateral, and radiolucent ureteral stones, solitary kidney, pregnancy, severe hydronephrosis, benign prostatic hyperplasia, renal insufficiency, a history of previous surgery, calcium channel blockers, or alpha-blockers therapy, severe hypertension (patients receiving more than 1 drug for hypertension), and refusal of MET.

### Workups and Measurements

Detailed physical examination, urine analysis, urine culture and sensitivity, blood urea and serum creatinine, abdominal ultrasound, kidney-ureter-bladder radiography (KUB), and non-contrast computed tomography (NCCT) were done for every patient. Age, sex, body mass index (BMI), laterality, and degree of hydronephrosis were recorded.

**Figure 1.**
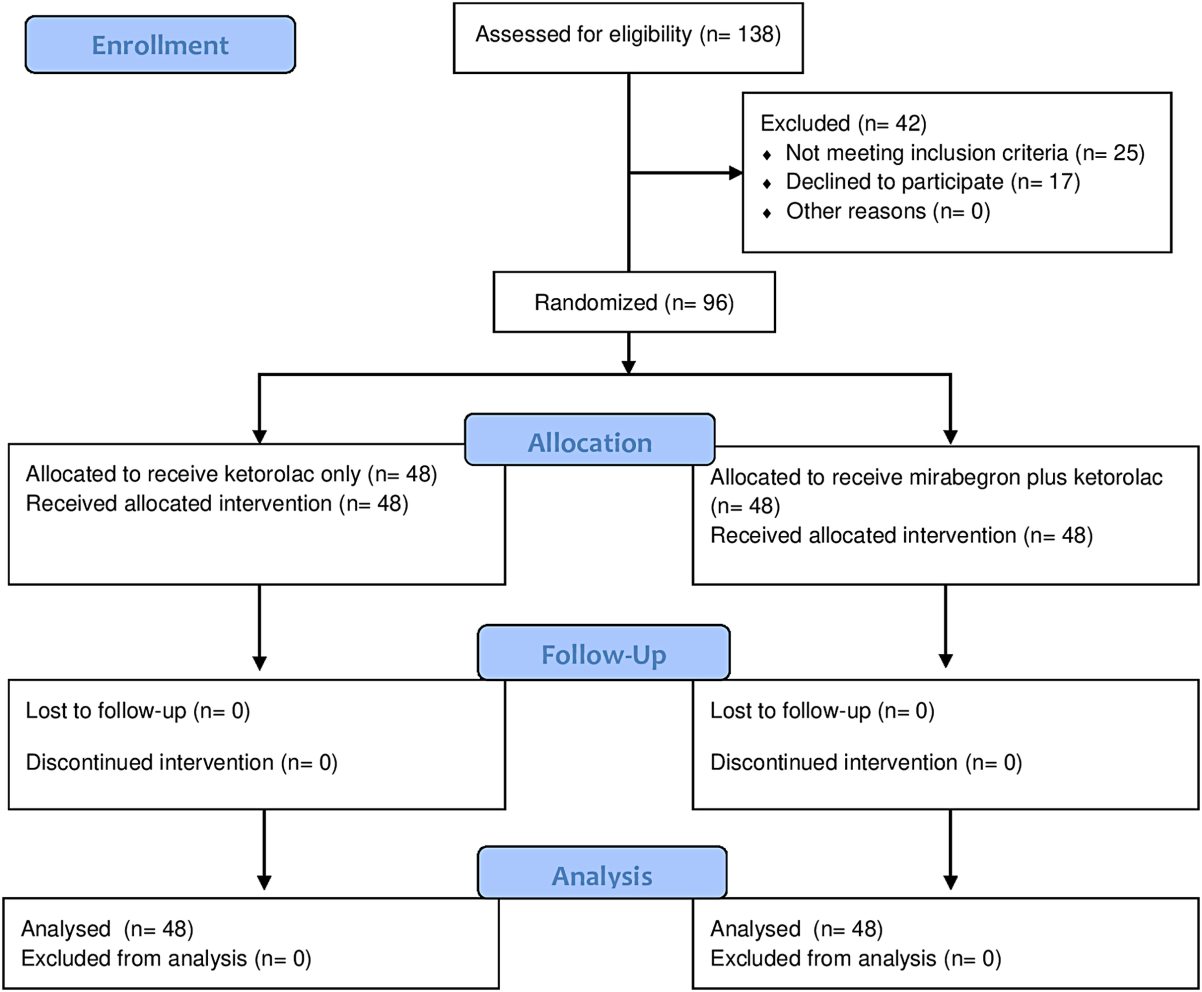
According to the Consolidated Standards of Reporting Trials (CONSORT) guidelines, a flowchart shows patients treated for distal ureteral stones of 5-10 mm by ketorolac only or mirabegron plus ketorolac.

We followed the Consolidated Standards of Reporting Trials guidelines to write this randomized trial (Figure 1). According to the randomization results and patients’ allocation schedule, group A included the patients who received only injectable ketorolac of 30 mg/day for 5 days and then on demand for a total of 4 weeks. Group B included the patients who received mirabegron of 50 mg/day for 4 weeks plus ketorolac 30 mg/day as in group A.

All patients were scheduled for weekly follow-up from the start of treatment. Abdominal ultrasound and KUB were done during every visit. After 4 weeks, patients in both groups were evaluated for stone expulsion (NCCT was used for those patients not documenting witness of stone passage), time to stone expulsion, pain episodes, analgesic usage, and intervention by ureteroscopy for those with failed stone expulsion. All patients needed to have their blood pressure measured daily during the study period. The primary outcome was defined as the stone expulsion rate (SER) at 4 weeks from the start of MET. Treatment failure was defined as the persistence of the stone in NCCT after 4 weeks. The secondary outcomes included the rates of renal pain episodes, time to stone expulsion, and needed extra analgesia during 4 weeks of MET.

### Ethical Considerations

Written informed consent was obtained from all patients for the plans of treatment and participation in this study. We followed the principles of the newest version of Helsinki Declaration with approval from the Ethics Committee of Assiut University Faculty of Medicine (17300684/2019).

### Statistical Analysis

Statistical analysis was performed using EasyMedStat software (version 3.15.1; www.easymedstat.com). Data were expressed as median (range; interquartile range) for quantitative data or number and percentage for qualitative data. The Mann–Whitney *U* test was used to compare groups in quantitative data, and the chi-squared test was used to compare groups in qualitative data. Multivariate logistic regression was performed to assess the relationship between SER and the explanatory variables: age, BMI, laterality, degree of hydronephrosis, stone size, and MET modality. Data were checked for multicollinearity with the Belsley–Kuh–Welsch technique. Heteroskedasticity and normality of residuals were assessed by Breusch–Pagan test and Shapiro–Wilk test, respectively. A *P*-value < .05 was considered statistically significant.

## Results

There was no statistically significant difference between the 2 groups in terms of age, sex, BMI, laterality, stone size, and degree of hydronephrosis. Patients’ demographic and clinical characteristics are shown in Table 1.

After 4 weeks, SER was 52.1% and 89.6% in groups A and B (*P* < .001), respectively (Table 2). This means that 23 patients (47.9%) failed to pass their stones in group A versus 5 (10.4%) patients only in group B; all of them were treated by ureteroscopy.

In univariate analyses, the outcome parameters including SER and the averages of time to stone expulsion, number of renal colic episodes, and number of extra analgesics were significantly different between both groups (Table 2). Seven patients (14.6%) versus 5 patients (10.4%) in groups A versus B, respectively, needed opioid analgesia other than ketorolac doses.

In the univariate analysis also, the medians (median, range; interquartile range) of stone size for patients with (5.7, 5-8.5; 1.2 mm) and without (7, 5.9-7.6; 1.6 mm) stone expulsion at 4 weeks, respectively, were significantly different (*P* < .001).

In multivariate analysis, the modality of MET (*P* < .001) and stone size (*P* < .001) were independent predictors of SER at 4 weeks (Table 3).

In group B, only 1 patient (2.1%) had a high-grade fever (39.2°C) that was objectively documented on presentation at the Emergency Department. Immediately after admission and start of symptomatic treatment, the stone passed, and then the fever subsided spontaneously before any invasive intervention. No other complications could be objectively reported in both groups.

## Discussion

The recent studies report usually the well-known SER of DUSs such as 71%-98% for stones <5 mm and 25%-51% for 5-10 mm stones.^3,12^ Factors such as location, size and number of stones, associated UTI, ureteral stricture or spasm, and ureteral anatomy can predict the spontaneous SER.^6^ Medical expulsive therapy provides smooth muscle relaxation to dilate the ureter and reduce edema and spasm, facilitating stone expulsion.^11^ Recent advances in basic research provided a better understanding of ureteral function and pathophysiology which has helped in employing MET as a conservative treatment approach.^6,14^ No doubt that proper analgesia is another important factor in this conservative management strategy. The most commonly used drug groups for analgesia are the non-steroidal anti-inflammatory drugs and opioids. The former group has been the first choice for the management of renal colic. The routinely used agents are diclofenac, ketorolac, and ketoprofen. These agents are used variably due to the availability of a suitable form for rapid control of the renal colic.^12,15^ The combination of analgesics and ureter-dilating agents is expected to be more effective than the individual agents alone. This issue has been proven with alpha-blockers and other drug groups such as phosphodiesterase-5 inhibitors.^3,4,15^

Classic METs containing alpha-adrenergic blockers and calcium channel blockers have their own adverse effects due to their underlying mechanism of action. Patients using these agents may suffer from retrograde ejaculation, nausea, dizziness, palpitation, and orthostatic hypotension.^3,6,16,17^ From this point of view, new agents such as β3AR agonists have been introduced as MET. They were studied for efficacy and safety in terms of sexual and cardiovascular adverse effects.^11^ Previously, β3AR agonists have been proven as the most recent effective agents in the treatment of overactive bladder.^18,19^

Many studies have been carried out on different mammals with β3AR supporting the opinion that β3AR agonists could be used as a new treatment method for DUSs. The presence of the functional expression of β3AR was shown and it was suggested that they could play a role in ureteral peristalsis and other ureteral function.^14,20,21^ They confirmed the expression of β1, β2, and β3 adrenoceptors in both smooth muscle and urothelial layers of the whole ureter. Based on these findings and their previous role in the management of overactive bladder, β3AR agonists can indirectly affect the muscular tone.^14^

Clinically, a few clinical studies have been conducted to examine the efficacy and safety of mirabegron as MET agent in patients with DUSs so far.^4,11-13,22,23^ They have different epidemiological designs including randomized trials which have been used to compare the outcomes of mirabegron with different agents of MET including alpha-blockers such as tamsulosin or sildosin.^4,22^ Also, agents of nonsteroidal anti-inflammatory drugs alone were compared with their combination with mirabegron.^13,23^ Mirabegron has variably been identified as effective in terms of SER which has usually been assigned as the primary outcome.^4,12^

In our study, there was higher SER in the mirabegron group than in the control group (89.6% vs. 52.1%). This was similar to the study of Solakhan et al^12^ who reported SERs of 87.5% versus 52.5% in the study and control groups, respectively. Also, there were lower averages of time to stone expulsion and number of episodes of renal colic in the mirabegron group. Again, Solakhan et al^12^ have the same results.

Regarding the stones >5 mm, Tang et al^4^ and Solakhan et al^12^ found no significant effects from the mirabegron combination with tamsulosin or diclofenac in comparison to tamsulosin or diclofenac alone, respectively. Our results were not similar to this finding, which can be attributed to the different drug combinations. However, the current univariate and multivariate analyses revealed that stone size was a significant and independent predictor of passage of 5-10 mm DUSs. On the other hand, the effect of mirabegron as MET agent was also confirmed as an independent predictor of high SER of these stones.

For providing evidence-based proof for the efficacy and safety of mirabegron as a MET agent, we recommend conduction of larger studies that should incorporate different MET agents such as mirabegron, alpha-blockers, and phosphodiesterase inhibitors in double-blind, randomized, comparative trials.

Limitations of our study included the limited generalizability of the results, because it is single-center work. Also, missing the measurement of the ureteral wall thickness due to stone impaction hindered the comparison of effect of edema on SER. Moreover, the long-term effects of mirabegron could not be evaluated due to the uncertain benefits of the outcomes.

In conclusion, mirabegron seems to be a promising MET agent in patients with DUSs. It has high efficacy in the form of high SER which can supervene after 2-4 weeks. It reduces the time to stone expulsion, episodes of renal colic, and the need for analgesia. Also, it was safe with a low complication profile. Mirabegron-based MET and stone size were independent predictors of SER in patients with solitary 5-10 mm DUSs. Hence, mirabegron plus ketorolac seemed to be superior to ketorolac alone for DUSs.

## Figures and Tables

**Table 1.** Patient Demographic and Clinical Characteristics

Variables	Group A (n = 48)	Group B (n = 48)	*P*
Median (Range; Interquartile Range) or n (%)	
Age (years)	41 (19-66; 47)	43.5 (19-65; 46)	.728
Gender			
Male	33 (68.7)	35 (72.9)	.312
Female	15 (31.3)	13 (27.1)	
Body mass index (kg/m^2^)	24 (19.4-35.5; 2.7)	23.6 (18.2-33.3; 3)	.334
Laterality			
Right	21 (43.7)	23 (47.9)	.456
Left	27 (56.3)	25 (52.1)	
Stone size (mm)	5.9 (5-10; 2)	6 (5-10; 1.1)	.759
Hydronephrosis degree			
None or mild	18 (37.5)	15 (31.3)	.811
Moderate	30 (62.5)	33 (68.7)	

**Table 2.** Outcomes of Treatment in Both Groups

Variables		Group A (n = 48)	Group B (n = 48)	*P*
Median (Range; Interquartile Range) or n (%)	
Stone expulsion rate	At 2 weeks	19 (39.6)	31 (64.6)	.041
At 4 weeks	25 (52.1)	43 (89.6)	<.001
Time to stone expulsion (days)		14 (13-23; 1.5)	7 (3-16; 10)	.004
Episodes of renal colic		1 (0-2; 1)	1 (0-2; 2)	<.001
Extra analgesic ampoules		1 (0-7; 4)	0 (0-2; 0)	<.001

**Table 3.** Multivariate Logistic Regression Analysis of Predictive Factors of Stone Expulsion Rate in All Patients (n = 96)

Variables	Odds Ratio	*P*
Age	1.0 [0.97;1]	.812
BMI	1.14 [0.94;1.4]	.185
Laterality	1.48 [0.45;4.9]	.517
Degree of HN	0.53 [0.17;1.72]	.291
Stone size	0.28 [0.15;0.54]	< .001
MET modality	0.07 [0.02;0.28]	< .001

BMI, body mass index; HN, hydronephrosis; MET, medical expulsive therapy.
